# Microbiome-Driven Proline Biogenesis in Plants under Stress: Perspectives for Balanced Diet to Minimize Depression Disorders in Humans

**DOI:** 10.3390/microorganisms10112264

**Published:** 2022-11-15

**Authors:** Silva Vujanovic, Josko Vujanovic, Vladimir Vujanovic

**Affiliations:** 1 Hospital Pharmacy, CISSS des Laurentides, Université de Montréal, Montréal, QC J8H 4C7, Canada; 2 Medical Imaging, CISSS des Laurentides, Lachute, QC J8H 4C7, Canada; 3 Food and Bioproduct Sciences, University of Saskatchewan, Saskatoon, SK S7N 5A8, Canada

**Keywords:** microbiome, symbionts, endophytes, proline, amino acids, biogenesis, plant, crops, stress, food, diet, consumption, integrative science, imaging, human health, depression

## Abstract

According to the World Health Organization (WHO), depression is a leading cause of disability worldwide and a major contributor to the overall global burden of mental disorders. An increasing number of studies have revealed that among 20 different amino acids, high proline consumption is a dietary factor with the strongest impact on depression in humans and animals, including insects. Recent studies acknowledged that gut microbiota play a key role in proline-related pathophysiology of depression. In addition, the multi-omics approach has alleged that a high level of metabolite proline is directly linked to depression severity, while variations in levels of circulating proline are dependent on microbiome composition. The gut–brain axis proline analysis is a gut microbiome model of studying depression, highlighting the critical importance of diet, but nothing is known about the role of the plant microbiome–food axis in determining proline concentration in the diet and thus about preventing excessive proline intake through food consumption. In this paper, we discuss the protocooperative potential of a holistic study approach combining the microbiota–gut–brain axis with the microbiota–plant–food–diet axis, as both are involved in proline biogenesis and metabolism and thus on in its effect on mood and cognitive function. In preharvest agriculture, the main scientific focus must be directed towards plant symbiotic endophytes, as scavengers of abiotic stresses in plants and modulators of high proline concentration in crops/legumes/vegetables under climate change. It is also implied that postharvest agriculture—including industrial food processing—may be critical in designing a proline-balanced diet, especially if corroborated with microbiome-based preharvest agriculture, within a circular agrifood system. The microbiome is suggested as a target for selecting beneficial plant endophytes in aiming for a balanced dietary proline content, as it is involved in the physiology and energy metabolism of eukaryotic plant/human/animal/insect hosts, i.e., in core aspects of this amino acid network, while opening new venues for an efficient treatment of depression that can be adapted to vast groups of consumers and patients. In that regard, the use of artificial intelligence (AI) and molecular biomarkers combined with rapid and non-destructive imaging technologies were also discussed in the scope of enhancing integrative science outcomes, agricultural efficiencies, and diagnostic medical precisions.

## 1. Introduction

**Figure 1:**
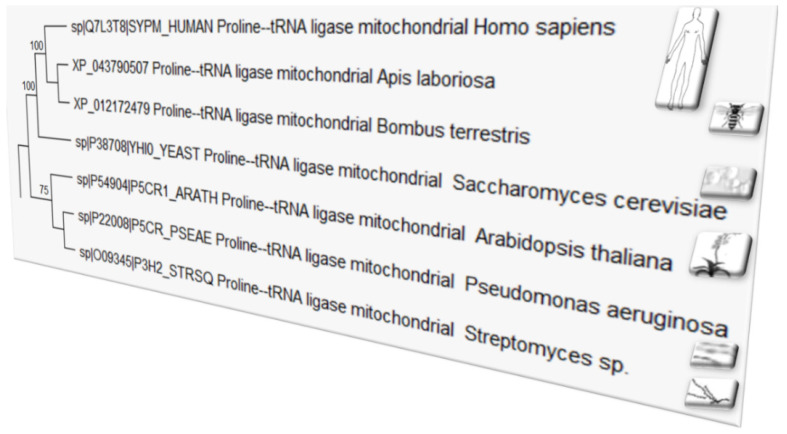
Phylogenetic tree based on maximum parsimony of the Proline-tRNA ligase, a mitochondrial enzyme that participates in proline metabolism, predicting the evolutionary history of taxa. (NOTE: Because of its central role in linking amino acids with nucleotide triplets contained in tRNAs, tRNA ligase is thought to be among the first proteins that appeared in evolution [5]). The evolutionary analyses presented in this phylogenetic tree were conducted in MEGA X [6,7].

**Figure 2:**
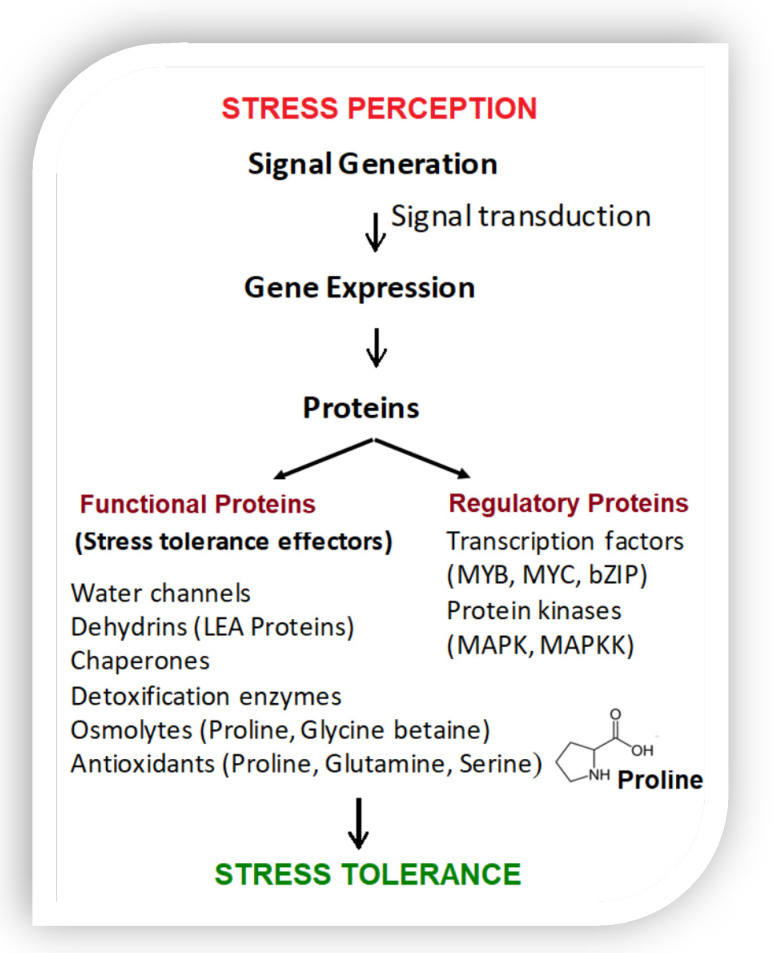
Relationship between stress resistance and proline in plants (data from Beck et al. [8]).

New molecular and metabolic studies have reported that a diet rich in the amino acid proline causes a severe state of depression in humans and animals including insects [1]. Amino acids are the building blocks of proteins, which are digested in the stomach into their constituent amino acids, resulting in improved body metabolism and functions. Among 20 different amino acids, proline is considered not essential, except in times of illness and stress. The coevolution theory of the origin of the genetic code has uncovered the importance of the proline biosynthetic pathway [2]. Indeed, this highly efficient osmolyte and antioxidant seems involved in the evolution of many organisms (Figure 1) due to its counteracting effect to multiple environmental stresses. In the model Arabidopsis, an intracellular proline level may increase by >100-fold, thus helping cellular metabolic stability under water deficit or salt-stress [3,4]. Proline accumulation in plants is a common physiological response to various stresses (Figure 2). It is usually enhanced by the endophytic microbial communities living inside healthy host tissues (seed, root, leaf, flower), playing an important role in mitigating abiotic and biotic stresses in plants.

Plant-associated microorganisms, such as endophytes, arbuscular mycorrhizal fungi (AMF), and plant growth-promoting (PGP) rhizobacteria, are well-documented for their role in promoting crop productivity and providing stress tolerance [9]. In staple food crops, the symbiotic plant microbiome positively affects plant water and nutrient acquisition, and stimulates flowering and fertility, fruit and grain production. The highest proline content across plant developmental stages occurs in plant generative tissues such as flowers, where nectar and pollen are produced in abundance [4]. Several insect species use the proline from plant food as a major energy substrate [10]; they show the ability to oxidize this amino acid at a high rate, which is considered a unique feature of this group of eukaryotes. The presence of proline in the haemolymph of bees and in the nectar of flowering plants led to the hypothesis that plants may produce proline as a metabolic reward for pollinators [11]. It appears that proline serves as metabolic fuel to power pollinators’ flights. Since honey is a natural food produced by honeybees from the nectar of flowers, it is not surprising that an extensive range in honey proline content ~300–900 mg/kg implies different flower origins [12]. Besides that, proline showed properties of a broad-based antioxidant, a trait which has been relatively unexplored in eukaryotic plant, fungi, insects, and mammalian systems [13]. In mammals and fungi, the potential of proline may be to suppress reactive oxygen species (ROS) and inhibit ROS-mediated cellular apoptosis [14]. To perform biological functions, the human body can biosynthesize some proline on its own from the amino acid L-glutamate. However, usually, humans and animals, including insects, boost proline levels by obtaining it directly from food sources.

## 2. Proline: Contradictory Roles in Healing and Excitotoxin Functions

Traditionally, the presence of proline was positively correlated with the functionality of the human body by helping form collagen, regenerating cartilage, forming connective tissue, curing skin damage and wounds, healing the gut lining, and repairing joints [15]. However, L-proline has been found to act as a weak agonist or activator of the glycine receptor and of both NMDA and non-NMDA (AMPA/Kainate) ionotropic glutamate receptors. Moreover, proline has been proposed to be a potential endogenous excitotoxin [16,17]. Excitotoxins are substances, usually amino acids, that overstimulate neuron receptors, similarly to chemical food additives such as monosodium glutamate. Neuron receptors facilitate brain cell communication and, upon excitotoxin exposure, overstimulate neurons in the brain [18]. This process, if prolonged, can exhaust and weaken the neurons involved, resulting in neuronal death. In short, excitotoxins can alter the chemistry of the brain to the point of no return. Recent findings revealed that a diet rich in proline was linked to an increased risk of depression in humans and animals [1], but there is a gap of knowledge regarding an equilibrated proline biosynthesis in plants and its accumulation in plant parts (in seeds/grains/nuts, flowers, fruits, and vegetables) used for a healthy diet. An efficient control of proline levels in preharvest agriculture and postharvest food and food commodities seems an inevitable step to undertake to prevent an excessive amount of proline entering plant-based foods, constituting a vital component of the overall human diet. Furthermore, the total amount of amino acids:proline ratio in plants and food might be a good indicator of the need to adjust proline dosage in human and animal diets. This is an area of science lagging behind in research in the field of food nutrients and functional foods. The idea is to prevent or minimize the negative impact of proline on mental health, i.e., to diminish proline-related neurological disorders, so particular attention must be paid to set a maximum dosage limit to prevent its excitotoxin effects such as depression in humans and animals.

## 3. Where Lie the Opportunities for Proline Science

**Figure 3:**
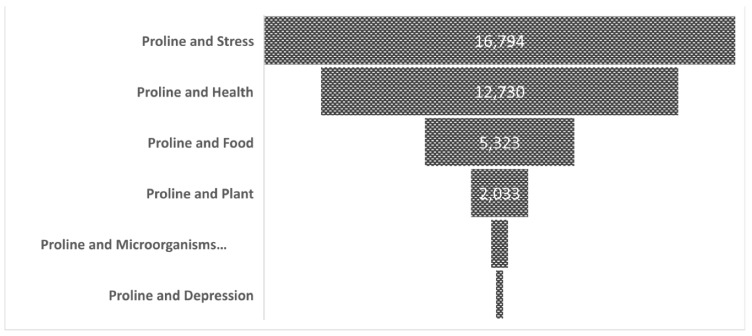
Web of Science data depicting published (1900–2022) articles on proline related to major scientific domains (data retrieved on 15 July 2022).

Publication survey metrics within proline science (Figure 3) is a critical step in identifying knowledge gaps that need to be filled to advance this emerging discipline. Currently, there is a total of about 35,000 publications on proline in the scientific literature. Web of Science data (1900–2022) suggest that Proline and Stress and Proline and Health topics largely prevail, together encompassing more than 80% of published papers or reports. Proline and Food comprises ~15% of publications. Proline and Plants comprises ~7% of publications, where there is mainly emphasis on the impact of proline on augmented plant resistance against environmental stresses, in particularly in the context of agriculture-related climate change issues [19]. In contrast, reports on the overall Proline and Microbiome and Proline and Depression subjects are marginal, together representing ~1% of published papers (Figure 3). Hence, the direction of scientific studies on proline should include a greater emphasis on microbiome-based approaches specific to plant genotypes under climate change and various stressors in different geographic locations.

**Figure 4:**
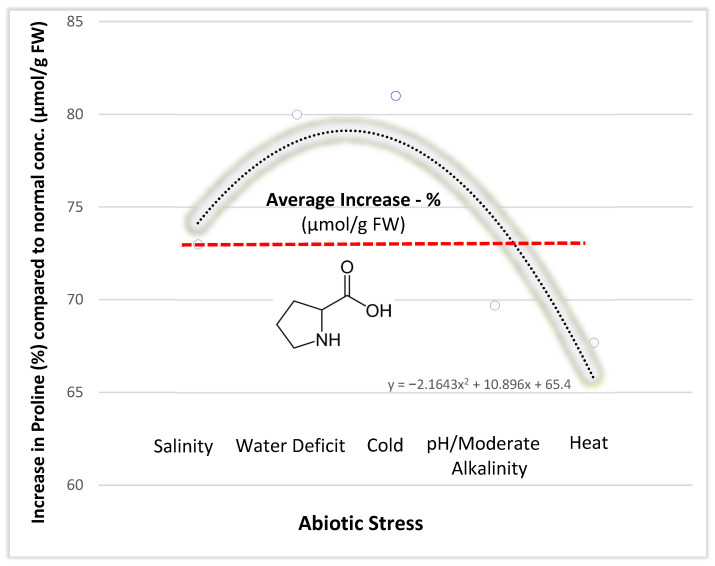
Summarizing data from recent Web of Science (1980–2020) articles on the average proline accumulation in staple wheat crops induced by individual abiotic stresses/climate-change-associated factors such as water deficit, salinity, pH, cold and heat. (Data retrieved on 10 June 2022).

A topic of particular interest for future science led by plant breeders, microbiologists, mycologists and entomologists who are interested in plant resistance, plant growth promotion and pest control, is to relate proline dynamics and translocation with microbiome-plant–host interactions [20] when exposed to biotic and/or abiotic stresses [21]. Nevertheless, establishing a basis for healthy food and feed, plant, grain, and vegetable products to meet new food and nutrition standards should include determining a threshold for an equilibrated proline content. Indeed, some reports indicate toxic effects of proline at higher concentrations as immensely induced in staple plants when exposed to various abiotic stresses [22,23]. Progress has been made in recent years regarding proline involvement and concentration in plants grown under a plethora of different abiotic stresses, without necessarily considering the microbiome component (Figure 4).

Proline is an excellent osmoprotectant in terms of healthy plant growth under salt stress [23]. Salinization is seen as a universal abiotic stressor, and it is used in most cultivated plant testing for comparison with other stressors with noticeable effects on nutrient deficiency, pH and oxidative stress. Its effect translates to biomass and crop yield decrease, and diminished quality of food [24]. In Figure 4, plants exposed to high salinity showed an increased average value (%) of proline concentration compared to other plant stressors. In plants, salt induced an average value of proline below that of high-water scarcity (drought) and cold, but this value lies above that induced by pH/alkalinity and heat stresses reviewed by previous papers [25,26,27,28,29,30]. Regarding proline distribution in plants, findings indicated a long-distance transport of proline in plant organs or tissues as critical for stress tolerance. Though proline biosynthesis occurs in cytosol and chloroplasts, proline is detected both in xylem and phloem, inferring its circulation through different tissues—particularly to pollen grains, thus enhancing plant fertility and thereby limiting seed loss [31] that greatly affects crop yield. 

At the cellular level, the proline osmolyte permits cell osmotic adjustment, stabilizes the structure of proteins and cell membranes, acts as a protective agent for enzymes, and is a free radical scavenger and antioxidant [32]. Proline can also provide regulation of cytosolic acidity, serves as a carbon and nitrogen reserve after stress relief, and may act as a signalling molecule able to activate defence responses [33]. In response to various environmental stresses, many plant species synthesize proline in the cytosol and accumulate it in chloroplasts [34], while ensuing shifts in cellular hormonal activities. The changes in endogenous hormonal balance were concomitant with accumulation of ROS and proline, accompanied by a loss of reducing potential [NAD(P)H/NAD(P)(+) and GSH/GSSG]. SA pretreatment scavenged drought-induced superoxide anion (O^2−^•) accumulation (but not H_2_O_2_) and led to an additional proline accumulation with enhanced expression of proline synthesis-related genes (P5CS1, P5CS2 and P5CR) and NADPH oxidase, and led to a reset of reducing potential with enhanced expression of redox regulating genes (TRXh5 and GRXC9). SA-mediated stress responses coincided with the enhanced expression of NPR1 and PR-1, with an antagonistic depression of ABA- and JA-related genes (NCED3, MYC2, and PDF1.2) [35]. These results indicate that the SA-modulated NPR1-dependent signaling pathway and proline synthesis represent an integrative process of redox control observed under drought. However, stress-induced proline accumulation is not a passive but rather a very dynamic process, as observed in Arabidopsis in low water potential polyethylene glycol (PEG)–agar plate assays [36]. In fact, seedlings transferred to low water potential caused a nearly 100-fold increase in proline content over 4 days. Proline increase during stress has helped plants to recover, and when the environment returned to a high-water potential, proline concentration returned to its initial level under a similar timeframe. This is clear evidence that proline turnover can buffer cellular redox status during drought. Proline synthesis and catabolism are regulated by multiple cellular mechanisms, of which we know only a few [37].

Overall, an increasing amount of evidence suggests that proline–plant interactions and kinetics are involved in plant stress tolerance at the species, organ, tissue, cellular and molecular levels. Determination of the relationship between stress resistance and level of proline in plants during all growth phenophases would play a preponderant role not only for future food security linked to a higher crop yield but also in controlling proline accumulation in food for a healthy diet. This cannot be easily achieved without knowledge of plant–microbiome interactions and their effects on the dynamics of proline metabolism in plants [37], as both proline and endophytic microbes are circulating within the plant tissues. Additionally, more scientific attention should be paid to integrated knowledge on the interconnection between plant health and human health topics. Plant-associated bacterial and fungal communities, similarly to human gut microbiota, increase host fitness under abiotic stresses, such as low temperature [38] and shifts in osmolarity [39,40], by stimulating proline metabolism pathways. In addition, host-associated symbiotic microorganisms accumulate important amounts of proline in their cells as a response to increased external osmolarity and loss of water [36,39,41,42]. A study of both proline accumulation and activation would garner some interesting and perhaps unexpected findings, as a microbiome influences insects’ and mammalians’ behavior throughout food consumption and digestion. The bidirectional plant genotype–microbiome interactions and proline biogenesis and decomposition may each affect individual-constituent species and assemblage of species, as these are modulated by environmental stimuli and availability of food.

## 4. Proline: Microbiome–Plant Axis

The plant microbiome is defined as a plant’s second genome [43] composed of a dynamic community of microbes. The host-associated prokaryotic and eukaryotic microbes are the biological foundations of every species and every ecosystem on the earth. Previous research mainly investigated the ecology and evolution of microorganisms associated with plants throughout environments, while contemporary research has shifted to better understand how microbial communities respond to a particular stress or a combination of stresses, which is notably important in meeting global agri-food demands. The aim is to manage plant–microbe interactions by creating stable plant ecosystems and a resilient phytobiome [44] for improved food security and safety [45]. The goal of this review study was to better understand the host and its microbiome and the role of the holobiont [46] and its functioning, as a plant genotype armed with its second genome or microbes may greatly affect proline production and metabolism in plants under multiple environmental stimuli. The idea is to come up with a proposition of more efficient preventive measures to minimize proline accumulation in plants, food and feed—as main sources of diets for human and animal consumption. Focusing on plant–microbiome mechanisms and dietary proline may open new avenues for innovative approaches in preventing depression in humans.

Recently, Mayneris-Perxachs et al. [1] revealed that a diet rich in proline is linked to microbiota alterations in the human gut system shifting to a proline metabolism, with possible impacts on depression. The proline levels in an animal or insect body depend on the host–microbiota profile, where increased presence of Lactobacillus coincides with less depression. However, the food chain starts with food produced from photosynthetic plants and ends with food digestion in the gut. Thus, the proline amino acid level in our body must also depend on proline levels in the diet, coming from symbiotic plants since harvested grains and vegetables are used for food and feed. Like the microbiome–gut–brain axis as a novel target in studying depression—a mental disorder with low treatment efficacy—the endophytic microbiome–plant (root and seed) axis also emerged as a target of research, with plant prenatal care as a strategy to alleviate plant stress. This concept nourishes innovations in agri-food systems [20]. In the same vein goes the need to propose an efficient measure to mitigate proline in field plants, while both microbiome-based farming and circular agriculture are gaining momentum.

**Figure 5:**
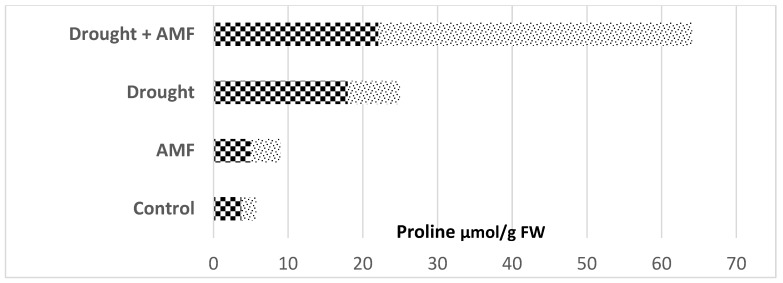
Symbiotic mycorrhizal fungi alone and abiotic stress alone, or their combination, altered accumulation of proline in 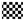 macadamia nut trees (*Macadamia tetraphylla* L.) and 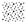 cucumber (*Cuccumis sativus*) plants (data from Hushem et al. [48] and Yooyongwech et al. [50]).

**Figure 6:**
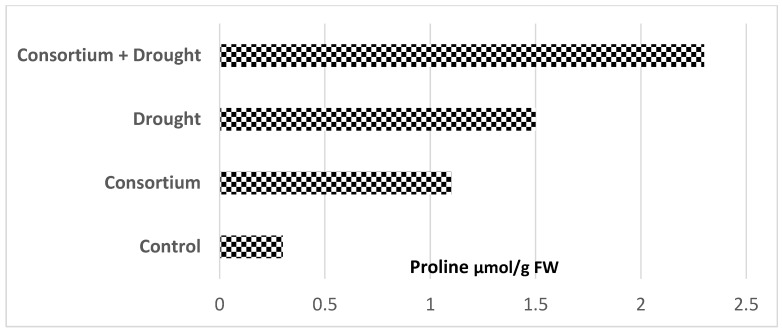
Plant-growth-promoting (PGP) Bacillus bacteria consortium (*B. subtilis*, *B. megaterium*, and *B. thuringiensis*) and abiotic stress alone, or their combination, altered average accumulation of proline in two different 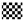 chickpea (*Cicer arietinum* L.) cultivars (data from Khan et al. [49]).

Plant–microbiome science is dominated by studies focusing on changes in host agricultural traits under drought, a major global single climate stress factor [47]. The story of the amino acid proline is fascinating due to its efficacy in enhancing plant tolerance to drought. In fact, beneficial plant microbiomes such as symbiotic mycorrhizal fungi (AMF) (Figure 5) and plant-growth-promoting (PGP) rhizobacteria (Figure 6) greatly contribute to increased proline concentrations in well-watered (control) plants [48,49,50]. Individual fungal or bacterial plant infections have been shown to trigger less proline concentration compared to drought stress in plants. However, both symbiotic fungi and bacteria, when combined with drought stress, showed a drastically increased proline accumulation (>70–90%) in plants, when compared with plants exposed to biotic (fungi and bacteria) or abiotic (drought) stress alone. The concentration trend of this metabolite remains unchanged when tested in different crop varieties (Figure 5).

Contemporaneous observations from cellular systems are particularly informative in terms of the role and regulation of proline metabolism. The fungal cells, compared to bacterial cells, accumulate less proline. It has been reported that symbiotic AMF and non-AMF contain less proline compared to the Bacillus–PGP species complex, i.e., ~0.5–1.0 mg/g FW [51] versus ~1.0–2.0 μmol/g FW [49], respectively. In yeasts, the mitochondrial energy metabolism is maintained through oxidative degradation of proline, and this process is important in regulating the longevity of fungal cells [52]. Bacteria and fungi often accumulate proline in response to increased external osmolarity and loss of water [36,38,42]. However, proline contributes to the pathogenesis of various disease-causing organisms and host–disease interactions [21]. An increasing number of studies show that proline acts as an antioxidant and suppresses apoptosis by regulating intracellular metabolite levels in microbial pathogens [14,53]. Thus, understanding the mechanisms of how pathogens utilize proline is important for developing new strategies against infectious diseases [21] and excessive proline formation in host plants.

**Figure 7:**
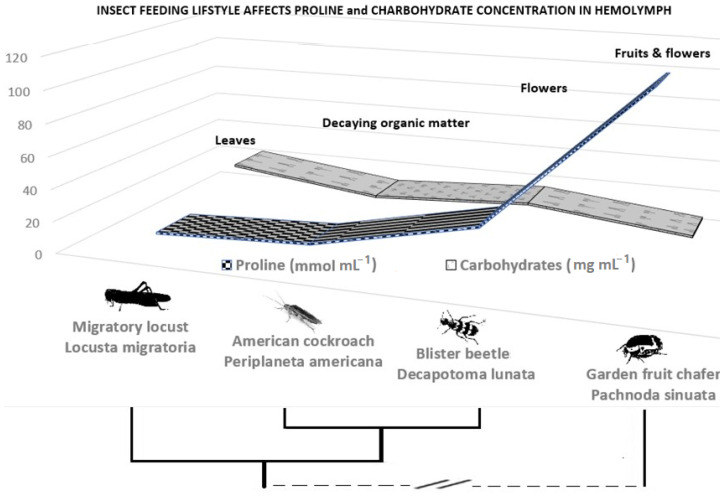
Shifts in concentrations of proline vs. total carbohydrates in the haemolymph of insects—based on insect’s feeding lifestyle and energy substrate (data from Auerswald and Gäde [54]).

From a system biology standpoint, the ever-growing interest is to picture the interactions not only between plants and microbiomes within changing environments but also to study associated insects acting as plant pests or vectors. According to Auerswald and Gäde [54], differences in plant organ and tissue types consumed by insect species determined the level of proline needed for insect lifecycle (Figure 7). The energy of substrates consumed and insect feeding lifestyle makes proline concentration higher in insects feeding on flowers and fruits compared to those feeding on leaves and dead organic matter (Figure 7). Under water deficit conditions, the root in sugar beet reduced by 1/3 in proline concentration compared to the leaf [55]. It appears that insects consuming flowers and fruits risk accumulating more proline and proportionally less carbohydrates in their bodies. Figure 7 shows that proline increased with decreased concentration of carbohydrates in insect hemolymph. Proline and sugars are important solutes that plants accumulate as osmoregulators when tissues are subjected to dehydration [56]. Their presence is inversely proportional in the body of different phylogenetic groups of insects. Interestingly, in honey, as a honeybee product, it was also reported that proline content decreased gradually with an increasing amount of added sugar products in all examined honey types [57].

Migratory locusts (*Locus migratoria* L.) show a relatively low proline content (Figure 7) and do not exhibit a significant increase in respiration rate due to external stimulation by proline. Conversely, hymenopterous bumblebee (*Bombus impatiens* Cresson) and wasp (*Vespula vulgaris* L.) species can oxidize proline, and more than double their respiratory capacities when proline is combined with carbohydrate-derived substrates [11]. The honeybees (*Apis mellifera* L.) show a relatively low increase in respiration with addition of proline compared with other hymenopterans.

Together, the findings of Teulier et al. [11] demonstrate that some bee and wasp species can greatly enhance the oxidation of carbohydrates using proline as fuel for flight. However, there is no evidence suggesting that gut microbiomes [38,58] and symbionts [59] can affect proline biosynthesis in insects—including in the *L. migratoria* model insect, as it was discovered within the human gut microbiome [1,60]. Stability of the insect gut as a microbial habitat is dependent, among other things, on the epithelial cells protected with secretory/gel-forming mucins [61] which also contain proline [62]. It would be worth exploring comparative insects’ microbiomes and associated biosynthesis of proline to make a better prediction of proline circulation within insects, plants, and microbes within a phytobiome level food chain. In any case, the case of migratory locusts’ low proline profile opens new scientific venues in the search for edible insect-based food and feed [63].

## 5. Future Perspectives

The observed changes in the taxonomic composition and diversity of the gut microbiota define the proline levels associated with brain damage severity and can be a biomarker of post-traumatic neurological deficit [60], as well as the state of depression in humans and animals [1]. Interestingly, proline concentration in eukaryotic plants and insect hosts is also regulated by microbial diversity and structure, altering internal biosynthesis and external acquisition of this amino acid. Consequently, host lifestyle and functionality, including feeding on energy substrates (food and feed) is related to proline circulation, with specific roles of host–microbiome-dependent activation or disruption of proline accumulation. Proline manipulation or level modulation is controlled by protocooperative host–microbe symbiotic interactions, where there is a protective role against excessive reactive oxygen species (ROS) formation in plant–host tissues or in human–host brain cells—i.e., a neuroprotective role in humans (e.g., against depression). Along with those protocooperative effects, adaptive shifts in microbial diversity and their metabolic profiles were observed. Supplementation of beneficial microbiomes targeting equilibrated proline biosynthesis and translocation at the cellular, tissue, organ and body systemic levels offers promising solutions to secure desirable proline levels in diets and improve proline digestion in preventing host disorders.

Mycorrhizal (AMF)-mediated low proline accumulation has been recorded in various plants under water deficit such as *Trifoliata*, *Citrus*, *Poncirus*, *Macademia* and *Cicer* [52,64,65,66], to name a few. Leguminous plants accumulated significantly less proline when associated with *Funneliformis mossae* (syn. *G. mosseae*) than with PGP inoculant *Bradyrhizobium* sp.–a root nodulating bacterium [66]. The mechanisms associated with lowering proline in symbiotic plants are poorly known. However, protocooperation in those healthy plants under stress conditions might functionally compensate for the need for proline. Current hypotheses consider different mechanisms, including the integration of proline synthesis inhibition and increased proline degradation in host cells, and AMF colonization-related increase in soluble sugar and non-structural carbohydrates (NSC) distribution through plant tissues. However, AMF showed some limitations in shifting accumulated proline levels through various mycorrhizal species-plant host combinations. Some symbiotic plants clearly exhibit important fluctuation in efficacy, going from increased to reduced proline concentration, when inoculated for example with *Rhizophagus intraradicens* compared to with *R. fasiculatus* or *Funneliformis mossae* or *Paraglomus occultum* (reviewed in [67]). Additional challenges in applying AMF species include low root colonization efficacy, particularly in Triticae cereals—major staple foods that provide key nutritional elements to the human diet. Indeed, the plant growth response in 27 tested wheat cultivars inoculated with mycorrhiza *R. intraradicens* varied from –36% to +19% [68].

Furthermore, emerging mycological discoveries on endophytes offer promising solutions. The next-generation fungal endophytes are vital symbiotic providers of multiple benefits to plants. Besides lowering proline in plants under drought stress, *Paraconiothyrium* SMCD 2210, for example, provides plant protection by inducing beneficial changes in microbiome communities in host plants and associated metabolomes to harness the host’s defense system, alleviate stress effects, and control insects [69]. It might mitigate proline metabolism and as such reduce the pathogenesis of various disease-causing organisms. In other words, inhibiting proline metabolism and transport may be a useful therapeutic strategy against some pathogens and insect pests [21]. The study on *Paraconiothyrium* SMCD 2210 highlights endosymbiotic plant growth promotion and alleviation of abiotic stress and ROS accumulation in germinating pea and chickpea seeds in plants grown under drought stress conditions, and so in addition to down-regulation of proline gene expression [70,71].

Chickpeas (*Cicer arietinum* L.) and peas (*Pisum sativum* L.) are two of the most important leguminous crops grown worldwide due to their nutritional and economic value. However, abiotic stress, primarily that induced by drought, limits legume production. Seeds of these two leguminous plants produced by F1 endosymbiotic plants under a controlled environment was used to conduct the second-generation (F2) growth study. Fungal and bacterial endosymbionts improved seed germination, colonization, and enhanced root and shoot growth in second-generation seeds produced by applying drought stress without endophytes. However, among several tested bacterial and fungal endophytes, only the SMCD 2210 strain down-regulated antioxidant proline gene expression for >100%, in addition to SOD-superoxide dismutase, manganese SOD-superoxide dismutase and dehydrin genes. These findings characterize enhanced oxidative stress tolerance and reduced reactive oxygen species (ROS) in symbiotic host cells, together with reduced proline. Like endophytes, some mycorrhizal (AMF) species also showed a similar tendency to moderate proline in stressed plants. The endosymbiont beneficial effect on plant resilience and improved phenotypes with reduced proline was translated into increased nutrient-protein quality of second-generation leguminous seeds [70,71]. These studies on leguminous plants indicate the potential of fungal endosymbionts to moderate drought-induced stress in plants by triggering epigenetic changes inherited across pea and chickpea generations, which correlate with enhanced resilience and improved agricultural traits in these globally important crops. These transgenerational effects of bacterial and fungal endophytes, named bactovitalism and mycovitalism, were also found in other staple crops such as wheat [72]. These plants experienced an enhanced biological seed stratification and germination and an early plant establishment [73], which implies multiple prenatal care effects in plants. In addition, FTIR spectroscopy analyses uncovered endophyte effects on shifts in plant-protective metabolic profiles against both abiotic (drought) and biotic (pathogen) stressors [74,75]. Recent reviews on plant symbiosis and plant microbiomes highlighted multiple opportunities offered by plant endophytes to global agri-food sectors [20,76].

## 6. Conclusions

Despite advancements in understanding the mechanisms of proline biosynthesis and metabolism, and its function in augmenting stress tolerance in procaryotes and eukaryotes, there are deficiencies in the overall understanding of the ecology of fungus–bacteria–insect–host–proline interactions and the way they have been investigated at the holobiont level. Although foods rich in proline may be linked to depression, it is unknown how to efficiently manipulate the proline concentration in plant- and food-based diets using the microbiome and thus to reduce the risk of depression in humans and animals.

A phytobiome approach that describes a holobiont within an environment offers an enormous potential for preventive control of proline accumulation in plants and food under climate change. The accumulation of this osmotolerant for crop survival under stress is often greatly enhanced in plants by a spectrum of symbiotic, endophytic, pathogenic, and commensal microbiota. In fact, the lack of knowledge and adequate measures to modulate proline levels raises concern as a plant-food-based diet can be a major issue related to depression in humans and animals. It appears that joint plant genotype–microbiome functionality affects proline in plants exacerbated with climate change, an additional confounding factor in tackling proline bioaccumulation in food and feed products. To address this problem at the source, we must focus on newly discovered plant endophytic symbionts that downregulate proline gene expression and prevent proline biosynthesis in plants under abiotic stress, while protecting plant health and global crops productivity. How is this feasible? Some fungal symbionts, such as endophytic fungi, and to a lesser extent mycorrhizal fungi and bacteria, are able to lower proline in both well-watered and -stressed plants through different mechanisms.

In this regard, and mainly based on a review of the literature supplementing our lab findings, the fungal endophyte effects on F1 plants and acquired epigenetic inheritance transmitted from parent to offspring should be taken into consideration to improve stress tolerance, together with ROS scavenging and reduced proline bioaccumulation in plants, while improving yield and grain nutritional value. The molecular mechanisms of proline-biosynthesis in stressed plants, although a complex issue, particularly when interacting on the phytobiome level with associated insects under a changing environment, is possible to control with symbiotic endophytes. This approach opens new opportunities for discoveries through combining research in the fields of agriculture, food nutrients and functional foods with the field of health science. Therefore, it is important to study the specificities of the protocooperative regulation of proline genes and proline biosynthesis in symbiotic plants, insects, and food products including honeys, using omics-innovative biotechnology integrated into plant–microbiome-based breeding programs designed to ameliorate crop traits across drought regions under climate change. The aim is to secure a diet without excessive proline accumulation. The effectiveness of this “green” strategy is inscribed in the control of this amino acid in the diet to prevent states of depression in humans and animals, including insects. It implies that newly acquired knowledge in pre- and post-harvest agriculture, including industrial food processing, may be critical to designing a proline-balanced diet.

**Figure 8:**
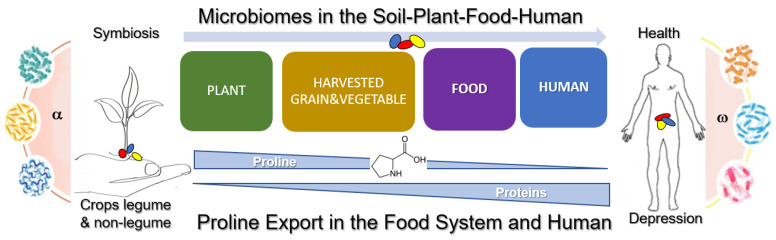
Holistic model for studying proline in the food chain aimed at controlling depression in humans and animals. Combining synergetic microbiome–plant axis with microbiome–human/animal/insect axis as a conceptual basis of targeting a more healthy diet.

Finally, we need a more holistic model system to study proline by combining the protocooperative microbiome–plant axis with the microbiome–human/animal/insect axis, targeting a healthier diet for humans and animals (Figure 8).

This synergetic agri-food and pharmaco-medical strategy within the complex landscape of mental health problems requires systems science [77] to build interdisciplinary bridges between diverse research fields in order to take better care of mental disorders globally. Attention should be paid to mycotoxin food co-contamination (trichothecenes, zearalenone, fumonisins, ochratoxins, and aflatoxins) in the presence of proline and how they affect the nervous system, directly or through immune cell activation [78], thus contributing to neuropsychiatric disorders [79,80] including depression. Mycotoxins specifically target high-protein-turnover and -activated cells, which are predominant in the gut epithelium [81]. In addition, some mycotoxins facilitate the persistence of intestinal microbial pathogens and potentiate intestinal inflammation [82]. Thus, mycotoxin ingestion may greatly affect gut microbiome composition and intestinal functions and metabolism, including proline systematic mobilisation through the human body. A better understanding of links between preharvest agriculture and postharvest food handling, and between mycotoxin exposure and excessive proline amounts entering plant-based foods, seems a vital component in understanding the overall human diet.

Further, plant and gut microbiome research is experiencing fundamental shifts, notably in ways that focus on analyzing large data quantities, typically to make better predictions about multiple causes and contributors to symptoms of depression. The use of artificial intelligence (AI) and molecular biomarkers in combination with imaging systems to increase agricultural efficiencies and diagnostic medical precisions is undergoing extensive evaluation. As such, microbiome research using AI and data science creates unique opportunities to personalize agriculture and medicine surrounding proline-related mental issues (Figure 8). The recent integration of molecular pathology epidemiology (MPE) [83], which allows a rapid progress in the health sciences of precision medicine and precision prevention, aims to improve precision public health globally. As such, the disturbative effects of diet on gut microbiota have been closely related to immunology and pathology, and can be readily integrated into MPE’s framework [84]. Practically, MPE research can conduct integrative analyses of biospecimens (plant, diet, and patient) considering both hosts’ endogenous and exogenous factors—including environmental, lifestyle, dietary, host germline genetics and microbial variations—and examine immunological and disease processes at the molecular, cellular, tissue, individual, and population levels [83].

Rapid visual and quantitative detection of proline and mycotoxins within biospecimens may help to evaluate the interplay between this amino acid and secondary metabolites, as well as their combined effect on neurological disorders, thus leading to better integrative understanding of depression epidemiology. Different radiological imaging techniques, such as magnetic resonance imaging (MRI) and dual-energy computed tomography (CT) scanning [84,85,86], can potentially be explored in assessing proline molecules in the human body, while nuclear magnetic resonance (NMR) and hyperspectral imaging (HSI) [87,88] have been studied in tracking proline and mycotoxins in plants and food. A future research goal may be to investigate the relation between shifts in environmental exposures, agricultural outcomes (e.g., crop yield, quality of agricultural products) and neurological findings. Diets can be a particular subject of meta-analyses in relation to any molecular mechanisms in plants or humans and any outcomes for preventing or reducing health risks. However, this type of approach is rarely taken. It could offer a wide opportunity to advance the science of depression disorders, which is currently underappreciated.

In this regard, digitalization and machine learning in agriculture science [89], as well as automation and deep neural networking in medical science [90,91], may provide complementary tools to researchers to enhance healthy food diet and related patient care. This may open new avenues for innovations and more effective depression treatments.
